# Marketing messages accompanying online selling of low/er and regular strength wine and beer products in the UK: a content analysis

**DOI:** 10.1186/s12889-018-5040-6

**Published:** 2018-02-08

**Authors:** Milica Vasiljevic, Lucia Coulter, Mark Petticrew, Theresa M. Marteau

**Affiliations:** 1 Behaviour and Health Research Unit, Institute of Public Health, Addenbrooke’s Hospital, University of Cambridge, Cambridge, CB2 0SR UK; 2 School of Clinical Medicine, University of Cambridge, Cambridge, CB2 0SP UK; 3 Faculty of Public Health and Policy, London School of Hygiene and Tropical Medicine, London, WC1H 9SH UK

**Keywords:** Marketing messages, Low/er strength alcohol, Online selling, Drinking occasions, Health-related claims

## Abstract

**Background:**

Increased availability of low/er strength alcohol products has the potential to reduce alcohol consumption if they are marketed as substitutes for higher strength products rather than as additional products. The current study compares the main marketing messages conveyed by retailers and producers for low/er and regular strength wine and beer products.

**Methods:**

A content analysis of the marketing messages stated (in text) or depicted (in image) for low/er and regular strength wines and beers sold online on the websites of the four main UK retailers (Tesco, ASDA, Sainsbury’s, and Morrisons), and the producers of these products between February–March 2016.

**Results:**

Four themes were identified: (a) suggested occasions for consumption, (b) health-related associations, (c) alcohol content, and (d) taste. Compared with regular strength products, low/er strength equivalents were more often marketed in association with occasions deemed to be suitable for their consumption including lunchtimes [wine: *X*^*2*^(1, *n* = 172) = 11.75, *p* = .001], outdoor events/barbeques [beer: *X*^*2*^(1, *n* = 96) = 11.16, *p* = .001] and on sport/fitness occasions [beer: *X*^*2*^(1, *n* = 96) = 7.55, *p* = .006]. Compared with regular strength wines and beers, low/er strength equivalents were more frequently marketed with images or text associated with health. These included images of fruit [wine: *X*^*2*^(1, *n* = 172) = 7.78, *p* = .005; beer: *X*^*2*^(1, *n* = 96) = 22.00, *p* < .001] and the provision of their energy (calorie) content [wine: *X*^*2*^(1, *n* = 172) = 47.97, *p* < .001; beer: *X*^*2*^(1, *n* = 96) = 15.10, *p* < .001]. Low/er strength products were also more often marketed with information about their alcohol content. There were few differences in the marketing messages regarding taste.

**Conclusions:**

Low/er strength wines and beers appear to be marketed not as substitutes for higher strength products but as ones that can be consumed on additional occasions with an added implication of healthiness.

**Electronic supplementary material:**

The online version of this article (10.1186/s12889-018-5040-6) contains supplementary material, which is available to authorized users.

## Background

Alcohol is the fifth leading cause of death and disability globally [1]. The cost of alcohol-related harms in the UK has been estimated at £21 billion a year, including costs to individual drinkers, to people around them, and to society [2]. The development, promotion and marketing of low/er strength alcohol products is proposed as one way of reducing alcohol consumption and associated harms [3]. This interest is captured in the most recent UK Government Alcohol Strategy published in March 2012 that, amongst other policies, included an industry pledge through the Responsibility Deal to take one billion units of alcohol out of the market by 2015, primarily through increasing consumer selection of low/er strength alcohol products [3]. However, it is disputed whether this pledge has been met [4].

For such products to reduce consumption depends on a number of assumptions. These include: first low/er strength alcoholic products being selected by consumers in place of higher strength products as opposed to simply increasing the number of opportunities perceived suitable for consuming alcohol (see also [5, 6]); and second labels highlighting low/er alcohol strength not engendering a self-licensing effect (i.e., giving people permission to consume more following what might be interpreted as a virtuous choice) resulting in the higher overall consumption of alcohol than would have been consumed from a higher strength product alone [7, 8]. Such effects may be mediated by the marketing messages used to sell low/er strength alcohol products.

There is a paucity of evidence regarding the marketing of such products to the general public. The purpose of the present study is to describe the main marketing messages used by retailers and producers of low/er strength wine and beer products. For the purposes of the study we defined low/er strength alcohol products as low alcohol products (i.e., less than 1.2% ABV including de-alcoholised products) and lower alcohol products (i.e., above 1.2% ABV but less than the rate at which duty rises - 8.5% ABV for wine and 2.8% ABV for beer). Regular strength alcohol products were defined as wines above 8.5% ABV and beers above 2.8% ABV. Marketing messages were defined as the messages stated in text or depicted in images in the online marketing materials on retailers’ and producers’ webpages (see [9]).

## Methods

### Aim

To describe the marketing messages stated (in text) or depicted (in image) by retailers and producers for low/er strength wine and beer products sold online by the four main supermarkets in the UK during February–March 2016, and to compare these messages with those used for comparable regular strength products.

### Sample identification

Searches were conducted of the websites of the four main UK retailers (Tesco, ASDA, Sainsbury’s, and Morrisons) between February and March 2016 to identify webpages marketing low/er strength wines and beers. For each webpage marketing low/er strength alcohol products the webpage of a comparable regular strength alcohol product was identified on the same website. Regular strength alcohol products which are as similar as possible to the identified low/er strength alcohol products were selected for analysis. For example, if a low/er strength rosé was identified in the initial search, we identified a similar rosé by the same producer with regular strength. If this was not possible, we selected a regular strength rosé from a different producer, made from the same grape.

Once all webpages marketing the low/er and regular strength alcohol products were identified on the four retailers’ websites, the website of each product’s producer was searched to identify the producer webpage marketing the product. In total, 86 webpages marketing 41 distinct low/er strength wines, and 48 webpages marketing 16 distinct low/er strength beers were identified. Correspondingly, 86 webpages marketing comparable regular strength wines and 48 webpages marketing comparable regular strength beers were identified.

Inclusion criteria were as follows:

- Marketing messages (both text and images) for (1) low/er and (2) regular strength wines and beers sold online on the websites of (a) the four main UK retailers (Tesco, ASDA, Sainsbury’s, and Morrisons); and (b) the producers of these products; and
- Text-based messages in English.

The marketing messages (both text and images) retrieved from websites meeting the above inclusion criteria were preserved as Adobe .pdf files which were shared between the two coders (MV, LC).

### Coding guide development and coding procedures

The first two authors independently pilot coded six products. A coding guide was developed after iteratively coding the marketing messages for these six products. The computation of inter-rater reliability served to establish reliability between the coders (Krippendorff’s *α* ≥ .67). Disagreements were resolved by discussion until perfect agreement was achieved. The first two authors used the guide to code the identified marketing messages for the presence or absence of the different coding features. Any disagreements in coding were again resolved after discussion.

#### Coding instrument

The following was recorded:

##### Website descriptions

Date of retrieval, uniform resource locator (URL) of website, and any age restrictions on website.

##### Product information

The types of products sold were coded as follows: low/er or regular strength wine, low/er or regular strength beer. The cost of the product was also recorded. Strength descriptors (e.g., “none,” “light,” “medium” and “high”) were recorded with the corresponding %ABV listed. Energy and calorie information were recorded too. Drinking occasions suggested on the webpage were also coded. Taste descriptors and the presence of different flavours were coded as present or absent.

### Analyses

The frequency of occurrence of each marketing message was calculated. After piloting the coding framework, the two coders iteratively reviewed and discussed examples of themes. Major thematic content areas were suggested following pilot analyses. A series of Chi squared tests were performed to compare differences in marketing messages of low/er vs. regular strength wines and beers. For all analyses, we considered a 5% Type I error rate at the level of the superordinate theme and type of drink (global), with a Holm-Šídák multiplicity correction which takes into account the dependence between the subordinate themes contained within a superordinate theme per drink (see [10]).

## Results

**Fig. 1:**
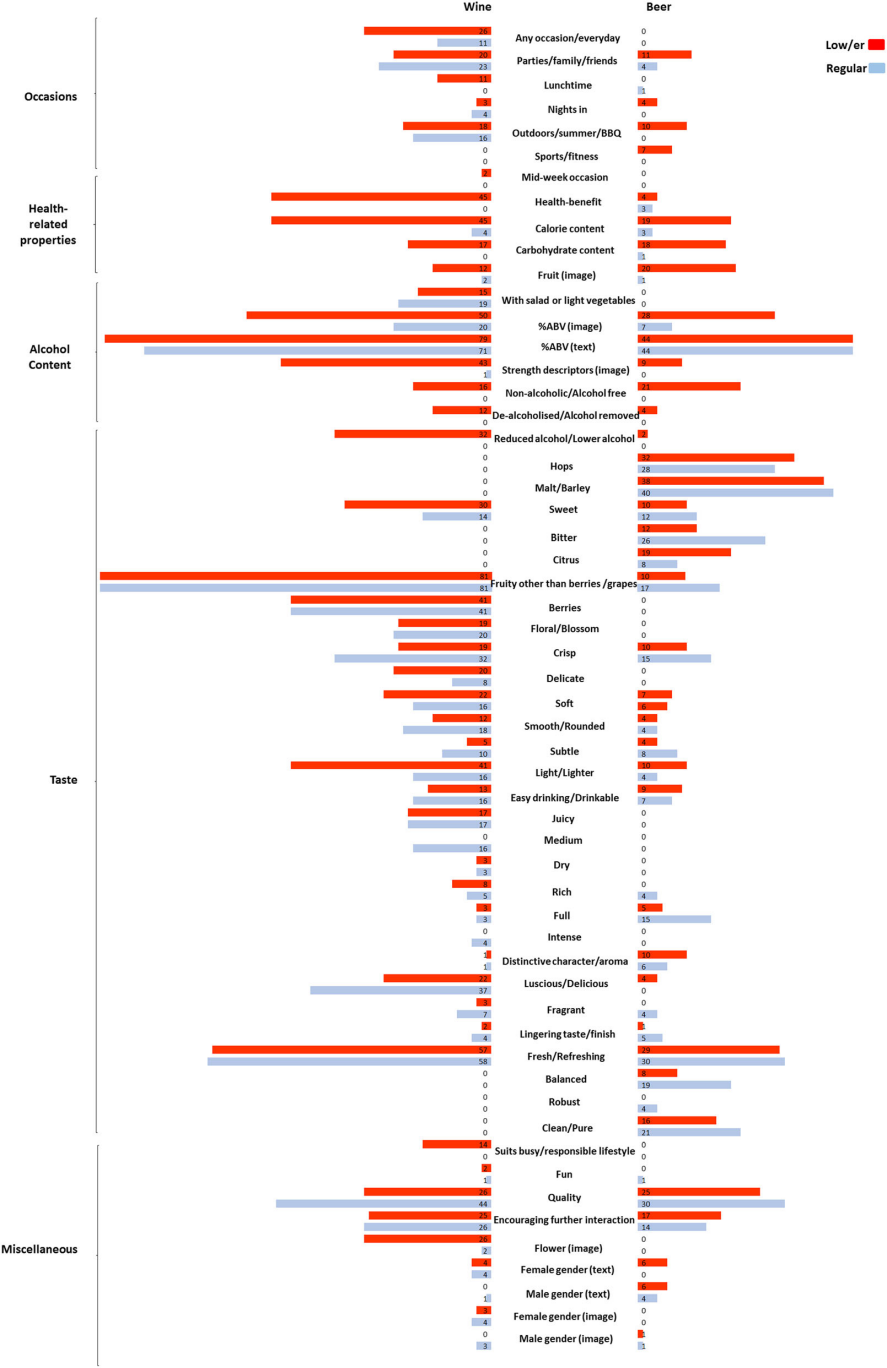
Graphical presentation of the frequency of appearance of marketing messages categorised under superordinate themes for low/er and regular strength alcohol products

Four themes were identified in the marketing messages: occasions, health-related associations, alcohol content and taste. Messages that did not fit within these four were categorised as “miscellaneous” (see Fig. 1 and Additional file 1: Table S1 in Online Supplementary Materials). Of these themes, the different occasion, health-related and alcohol content messages were more often present for the low/er strength alcohol products. For the “taste” and “miscellaneous” categories there were few significant differences in their presence between the low/er and regular strength products, and these contrasts did not reach the global significance level after correcting for multiple comparisons. These results suggest that taste descriptors may not be used to distinguish between low/er and regular strength wines and beers in marketing messages contained on producers’ and UK retailers’ webpages (more details on the taste and miscellaneous superordinate themes can be found in Additional file 1: Table S1 in Online Supplementary Materials).

Low/er compared to regular strength wines were significantly more likely to be marketed as suitable for consumption on any occasion/everyday [e.g., *‘Perfect option for every taste and occasion’*] [*X*^*2*^(1, *n* = 172) = 7.75, *p* = .005], as well as being suitable for consumption during lunch-times [e.g., *‘Perfect for all occasions from a lunchtime barbeque to an evening celebration’*] [*X*^*2*^(1, *n* = 172) = 11.75, *p* = .001]. Similarly, low/er strength beers were more likely to be marketed as suitable for outdoor/summer picnic/barbeques [e.g., *‘A perfect way to refresh whether it’s at a BBQ, down at the beach, or when you’re watching the game with mates’*] [*X*^*2*^(1, *n* = 96) = 11.16, *p* = .001], and for sports and fitness occasions [e.g., *‘To refresh thirsty sportsmen and women’*] [*X*^*2*^(1, *n* = 96) = 7.55, *p* = .006].

Low/er strength wines were more likely to include marketing messages associated with health [e.g., *‘Great if you're watching your weight but still hanker after a little bit of what you fancy’*] [*X*^*2*^(1, *n* = 172) = 60.95, *p* < .001], and information about calorie [*X*^*2*^(1, *n* = 172) = 47.97, *p* < .001], and carbohydrate content [*X*^*2*^(1, *n* = 172) = 18.87, *p* < .001]. Similar findings were observed for beer as regards to information about calorie [*X*^*2*^(1, *n* = 96) = 15.10, *p* < .001], and carbohydrate content [*X*^*2*^(1, *n* = 96) = 18.96, *p* < .001]. Low/er, compared with regular strength wines and beer, were more often marketed with images of fruits [wine: *X*^*2*^(1, *n* = 172) = 7.78, *p* = .005; beer: *X*^*2*^(1, *n* = 96) = 22.00, *p* < .001].

Low/er strength products were also more likely to contain messages regarding alcohol content: including %ABV information in their images [wine: *X*^*2*^(1, *n* = 172) = 21.68, *p* < .001; beer: *X*^*2*^(1, *n* = 96) = 19.83, *p* < .001], and strength descriptors such as “light” or “lower” visible in their images [wine: *X*^*2*^(1, *n* = 172) = 53.87, *p* < .001; beer: *X*^*2*^(1, *n* = 96) = 9.93, *p* = .002]. For a sample of verbatim marketing messages pertaining to occasions and health-related claims please see Table 1.

**Table 1:** Sample marketing messages for low/er and regular strength alcohol products for the two main superordinate themes identified: (a) occasions and (b) health-related properties

Superordinate theme	Marketing message	Sample verbatim text on wine webpages	Sample verbatim text on beer webpages
Occasions	Any occasion/ everyday	‘All occasions’ (L); ‘Any occasion’ (L, R); ‘Any time’ (L); ‘Whenever you want’ (L); ‘Perfect option for every taste and occasion’ (L); ‘Very drinkable whatever the weather’ (L); ‘You never need to feel left out, whatever the situation’ (L)	None
	Parties/family/friends	‘For lazy afternoons and relaxing with friends’ (R); ‘With your mates’ (L, R); ‘Get-togethers’ (R); ‘An evening celebration’ (L); ‘Dinner parties’ (L, R)	‘A perfect way to refresh whether it’s at a BBQ, down at the beach, or when you’re watching the game with mates’ (L); ‘For all your trendy patio parties, picnic classics and the good old-fashioned night in with your pals’ (L); ‘For those occasions when you need to smarten up a little’ (R)
	Lunchtime	‘Lunchtime treat’ (L); ‘Lunchtime tipple’ (L); ‘Perfect for all occasions from a lunchtime barbeque to an evening celebration’ (L)	‘Both dinner and lunch’ (R)
	Nights in	‘Perfect for nights in and social get-togethers’ (R); ‘Quiet night in front of the TV’ (L)	‘For all your trendy patio parties, picnic classics and the good old-fashioned night in with your pals’ (L)
	Outdoors/ summer/BBQ	‘Perfect for the warm days of summer or enjoyed year round’ (R); ‘Al fresco’ (R); ‘Barbecue’ (L, R); ‘Picnics’ (L, R)	‘A perfect way to refresh whether it’s at a BBQ, down at the beach, or when you’re watching the game with mates’ (L); ‘For all your trendy patio parties, picnic classics and the good old-fashioned night in with your pals’ (L)
	Sport or fitness	None	‘To refresh thirsty sportsmen and women’ (L); ‘Isotonic recovery drink’ (L)
Health-related properties	Health-benefit	‘X % fewer calories’ (L); ‘X % less calories’ (L); ‘Lower in calories’ (L); ‘Less calories’ (L); ‘With only X calories in a 125 ml glass’ (L); ‘You don’t have to give up on the Pinot Grigio when you’re cutting back on calories’ (L); ‘Diet friendly’ (L); ‘Who said dieting couldn’t be fun?’ (L); ‘Great if you’re watching your weight but still hanker after a little bit of what you fancy’ (L); ‘If you don’t fancy being full-bodied, it’s just the drop for you’ (L); ‘It’s only the wine that stays big and full bodied and you don’t’ (L); ‘No artificial colours or flavours’ (L); ‘With natural flavours’ (L)	‘Less gassy than many other lagers’ (R); ‘Natural ingredients’ (L); ‘Vitamin rich… isotonic thirst quencher… Provides the body with essential nutrients… Folic acid and vitamin B12 reduce fatigue and support the immune system. B12 also promotes energy-yielding metabolism. Just one bottle per day contributes to healthy nutrition’ (L)
	Calorie content	‘X calories’(L, R); ‘X kcals’ (L, R)	‘X calories’ (L, R); ‘X kcals’ (L, R)
	Carbohydrate content	‘X g carbohydrate’ (L)	‘X g carbohydrate’ (L, R)

*Note.* Sample marketing messages accompanying low/er strength alcohol products are denoted with an L in parentheses, and the regular strength alcohol products are denoted with an R in parentheses

## Discussion

Compared with regular strength products, low/er strength equivalents were more often marketed in association with occasions deemed to be suitable for their consumption including lunchtimes [wine], outdoor events/barbeques [beer] or on sports and fitness occasions [beer]. Furthermore, compared with regular strength wines and beers, low/er strength equivalents were more frequently marketed with images or text associated with health. These included images of fruit and the provision of their energy (calorie) and carbohydrate content.

Presenting low/er strength alcohol products as suitable for consumption on a wider range of occasions than regular strength products suggests they may be being marketed to replace soft drinks rather than alcohol products of regular strength. This raises a broader question of the extent to which low/er strength alcohol products will contribute to a public health strategy to reduce alcohol consumption [4, 6].

Furthermore, the marketing messages suggesting extended occasions for low/er strength alcohol consumption may be additional to regular strength alcohol consumption, while maintaining or extending recognition of the main brand in question. Importantly, although the development and sale of low/er strength alcohol alternatives has been portrayed by the alcohol industry as a way of removing units from the market (thereby reducing alcohol consumption in the population) [3], none of the marketing messages captured on retailers’ and producers’ websites marketing low/er strength alcohol wines and beers mentioned drinking less or reducing alcohol harms (see also [4, 11]).

The explicit reference to health benefits of low/er strength alcohol alternatives suggests that the industry and retailers may be targeting the health conscious “millenials” who now form a large portion of the drinks market (see [12]). It may also be part of a wider industry strategy to imply the health benefits of alcohol more generally which is currently not possible with regular strength products under existing advertising restrictions [13]. This is consistent with the current industry strategy to position alcohol consumption as “part of a healthy lifestyle” [11, 14, 15]. Finally, linking consumption of low/er strength beer with sports is reminiscent of the traditional mode of regular strength alcohol advertising which has often been synonymous with sponsorship of sports events [16]. This is concerning because the linking of alcohol with sports and fitness has been associated with risky drinking [17]. Low/er strength alcohol products may therefore be part of this wider industry strategy.

The study was limited in its sampling frame thereby limiting the extent to which we can generalise our findings beyond this. We only coded low/er strength wines and beers being sold in UK supermarkets. Furthermore, due to time and resource constraints, we only coded the low/er strength wines and beers found on the websites of the four main supermarkets in the UK. It is also important to note that other platforms such as billboards and social media (such as Facebook) may carry marketing messages that differ to those found in the present research which was limited in its focus on retailers’ and producers’ webpages. Future studies could usefully extend the sampling frame to include other marketing platforms. Furthermore, our sampling strategy used the levels at which duty rises as thresholds to define regular strength products (with any wine above 8.5%ABV and beer above 2.8%ABV defined as regular strength). Even though very few regular strength products were near this minimum threshold (with only one beer product at 3.8%ABV and a few wines at 9–9.5%ABV, with most products belonging to the regular category being of greater %ABV), it is possible that this choice of rather low minimum thresholds may have masked some of the differences between the marketing messages used for low/er and regular strength wines and beers, thus resulting in more conservative estimates of these differences.

Testing whether marketing messages on low/er strength alcohol products differed between producers espousing “responsible drinking” values and those producers not espousing such values was beyond the scope of this study. Future research could examine whether producers’ self-proclaimed “responsible drinking” values translate into the marketing strategies they ultimately pursue.

## Conclusions

Low/er strength wines and beers appear to be marketed not as substitutes for higher strength products but as ones that can be consumed on additional occasions with an added implication of healthiness. The present findings cast doubt on the industry contention that the development, promotion and marketing of low/er strength alcohol products may reduce alcohol consumption and associated harms [3]. Rather, the present findings add to an existing literature that highlights how measures intended to benefit public health (in this case wider availability of low/er strength alcohol products) may benefit industry to the detriment of the health of the public (see also [18]).

## Additional file

Additional file 1: Table S1. Frequency of appearance of the different marketing messages organised under the four superordinate themes and the miscellaneous theme for low/er and regular strength alcohol products. (PDF 293 kb)
