# Supplementary material for: Marketing messages accompanying online selling of low/er and regular strength wine and beer products in the UK: a content analysis
Source: BMC Public Health. 2018 Feb 8;18:147. doi: 10.1186/s12889-018-5040-6 (PMC5804076; doi:10.1186/s12889-018-5040-6)
Supplement: Additional file 1: Table S1. — Frequency of appearance of the different marketing messages organised under the four superordinate themes and the miscellaneous theme for low/er and regular strength alcohol products. (PDF 293 kb) [file 12889_2018_5040_MOESM1_ESM.pdf]

ONLINE SUPPLEMENTARY MATERIALS

ADDITIONAL FILE 1

Marketing messages accompanying online selling of low/er and regular strength wine and beer products in the UK: A content analysis

Milica Vasiljevic[a], Lucia Coulter[b], Mark Petticrew[c], & Theresa M. Marteau[a]

[a] Behaviour and Health Research Unit, University of Cambridge, UK

[b] School of Clinical Medicine, University of Cambridge, UK

[c] Faculty of Public Health and Policy, London School of Hygiene and Tropical Medicine, UK

Table S1 .... pp. 2-4

Table S1.
Frequency of appearance of the different marketing messages organised under the four superordinate themes and the miscellaneous theme for low/er and regular strength alcohol products.

		Wine			Beer		
**Superordinate theme**	**Marketing message**	**Low/er strength (n=86)**	**Regular strength (n=86)**	X^2 **(1, n=172)**	**Low/er strength (n=48)**	**Regular strength (n=48)**	X^2 **(1, n=96)**
**Occasions**	**Any occasion/ everyday**	26 (30%)	11 (13%)	7.75 (p=.005)*	0	0	-
	**Parties/family/ friends**	20 (23%)	23 (27%)	0.28 (p=.597)	11 (23%)	4 (8%)	3.87 (p=.049)
	**Lunchtime**	11 (13%)	0	11.75 (p=.001)*	0	1 (2%)	1.01 (p=.315)
	**Nights in**	3 (4%)	4 (5%)	0.15 (p=.700)	4 (8%)	0	4.17 (p=.041)
	**Outdoors/ summer/BBQ**	18 (21%)	16 (19%)	0.15 (p=.702)	10 (21%)	0	11.16 (p=.001)*
	**Sport or fitness**		-	-	7 (15%)	0	7.55 (p=.006)*
	**Mid-week occasion**	2 (2%)	0	2.02 (p=.155)	-	-	-
**Health-related properties**	**Health-benefit**	45 (52%)	0	60.95 (p<.001)*	4 (8%)	3 (6%)	0.15 (p=.695)
	**Calorie content**	45 (52%)	4 (5%)	47.97 (p<.001)*	19 (40%)	3 (6%)	15.10 (p<.001)*
	**Carbohydrate content**	17 (20%)	0	18.87 (p<.001)*	18 (38%)	1 (2%)	18.96 (p<.001)*
	**Fruits** (image)	12 (14%)	2 (2%)	7.78 (p=.005)*	20 (42%)	1 (2%)	22.00 (p<.001)*
	**With salad or 'light' vegetables**	15 (17%)	19 (22%)	0.59 (p=.444)	0	0	-
**Alcohol content**	**%ABV** (image)	50 (58%)	20 (23%)	21.68 (p<.001)*	28 (58%)	7 (15%)	19.83 (p<.001)*
	**%ABV** (text)	79 (92%)	71 (83%)	3.34 (p=.068)	44 (92%)	44 (92%)	0 (p=1.000)
	**Strength descriptors** (image)	43 (50%)	1 (1%)	53.87 (p<.001)*	9 (19%)	0	9.93 (p=.002)*
	**Non-alcoholic/alcohol free**	16 (19%)	0	17.64 (p<.001)*	21 (44%)	0	26.88 (p<.001)*
	**De-alcoholised/alcohol removed**	12 (14%)	0	12.90 (p<.001)*	4 (8%)	0	4.17 (p=.041)

	**Reduced alcohol/lower alcohol**	32 (37%)	0	39.31 (p<.001)*	2 (4%)	0	2.04 (p=.153)
**Taste**	**Hops**	-	-	-	32 (67%)	28 (58%)	0.71 (p=.399)
	**Malt /Barley**	-	-	-	38 (79%)	40 (83%)	0.27 (p=.601)
	**Sweet**	30 (35%)	14 (16%)	7.82 (p=.005)	10 (21%)	12 (25%)	0.24 (p=.627)
	**Bitter**	-	-	-	12 (25%)	26 (54%)	8.54 (p=.003)*
	**Citrus**	-	-	-	19 (40%)	8 (17%)	6.24 (p=.013)
	**Fruity/fruit (other than berries or grapes)**	81 (94%)	81 (94%)	0 (p=1.000)	10 (21%)	17 (35%)	2.53 (p=.112)
	**Berries**	41 (48%)	41 (48%)	0 (p=1.000)	-	-	-
	**Floral/blossom**	19 (22%)	20 (23%)	0.03 (p=.856)	-	-	-
	**Crisp**	19 (22%)	32 (37%)	4.71 (p=.030)	10 (21%)	15 (31%)	1.35 (p=.245)
	**Delicate**	20 (23%)	8 (9%)	6.14 (p=.013)	-	-	-
	**Soft**	22 (26%)	16 (19%)	1.13 (p=.288)	7 (15%)	6 (13%)	0.09 (p=.765)
	**Smooth/rounded**	12 (14%)	18 (21%)	1.45 (p=.228)	4 (8%)	4 (8%)	0 (p=1.000)
	**Subtle**	5 (6%)	10 (12%)	1.83 (p=.177)	4 (8%)	8 (17%)	1.52 (p=.217)
	**Light/lighter**	41 (48%)	16 (19%)	16.40 (p<.001)*	10 (21%)	4 (8%)	3.01 (p=.083)
	**Easy drinking/drinkable**	13 (15%)	16 (19%)	0.37 (p=.541)	9 (19%)	7 (15%)	0.30 (p=.584)
	**Juicy**	17 (20%)	17 (20%)	0 (p=1.000)	-	-	-
	**Medium**	0	16 (19%)	17.64 (p<.001)*	-	-	-
	**Dry**	3 (4%)	3 (4%)	0 (p=1.000)	-	-	-
	**Rich**	8 (9%)	5 (6%)	0.75 (p=.387)	0	4 (8%)	4.17 (p=.041)

	**Full**	3 (4%)	3 (4%)	0 (p=1.000)	5 (10%)	15 (31%)	6.32 (p=.012)
	**Intense**	0	4 (5%)	4.10 (p=.043)	-	-	-
	**Distinctive character/aroma**	1 (1 %)	1 (1 %)	0 (p=1.000)	10 (21%)	6 (13%)	1.20 (p=.273)
	**Luscious/delicious**	22 (26%)	37 (43%)	5.81 (p=.016)	4 (8%)	0	4.17 (p=.041)
	**Fragrant**	3 (4%)	7 (8%)	1.70 (p=.192)	0	4 (8%)	4.17 (p=.041)
	**Lingering taste/finish**	2 (2%)	4 (5%)	0.69 (p=.406)	1 (2%)	5 (10%)	2.84 (p=.092)
	**Fresh/ refreshing/ thirst-quenching**	57 (66%)	58 (67%)	0.03 (p=.871)	29 (60%)	30 (63%)	0.04 (p=.834)
	**Balanced**	-	-	-	8 (17%)	19 (40%)	6.24 (p=.013)
	**Robust**	-	-	-	0	4 (8%)	4.17 (p=.041)
	**Clean/Pure**	-	-	-	16 (33%)	21 (44%)	1.10 (p=.294)
**Miscellaneous**	**Suits busy/responsible lifestyle**	14 (16%)	0	15.24 (p<.001)*	-	-	-
	**Fun**	2 (2%)	1 (1%)	0.34 (p=.560)	0	1 (2%)	1.01 (p=.315)
	**Quality**	26 (30%)	44 (51%)	7.81 (p=.005)*	25 (52%)	30 (63%)	1.06 (p=.302)
	**Encouraging further interaction**	25 (29%)	26 (30%)	0.03 (p=.867)	17 (35%)	14 (29%)	0.43 (p=.513)
	**Flowers** (image)	26 (30%)	2 (2%)	24.57 (p<.001)*	-	-	-
	**Female gender** (text)	4 (5%)	4 (5%)	0 (p=1.000)	6 (13%)	0	6.40 (p=.011)
	**Male gender** (text)	0	1 (1%)	1.01 (p=.316)	6 (13%)	4 (8%)	0.45 (p=.504)
	**Female gender** (image)	3 (4%)	4 (5%)	0.15 (p=.700)	-	-	-
	**Male gender** (image)	0	3 (4%)	3.05 (p=.081)	1 (2%)	1 (2%)	0 (p=1.000)

Note. All comparisons denoted with a star [*] were statistically significant at $p < .05$ when correcting for multiple comparisons by using the Holm-Šídák correction at the level of each superordinate theme and type of drink.
